# Tumor‐Expressed SPPL3 Supports Innate Antitumor Immune Responses

**DOI:** 10.1002/eji.202451129

**Published:** 2024-12-10

**Authors:** Tamara Verkerk, Antonius A. de Waard, Sofie J. I. Koomen, Jasper Sanders, Tineke Jorritsma, Anouk T. Pappot, Nordin D. Zandhuis, Tao Zhang, Manfred Wuhrer, Arie J. Hoogendijk, Floris P. J. van Alphen, Maartje van den Biggelaar, Hannes S. J. Stockinger, Klaas P. J. M. van Gisbergen, Robbert M. Spaapen, S. Marieke van Ham

**Affiliations:** ^1^ Department of Immunopathology Sanquin Research Amsterdam The Netherlands; ^2^ Landsteiner Laboratory, Amsterdam UMC, University of Amsterdam Amsterdam The Netherlands; ^3^ Department of Hematopoiesis Sanquin Research Amsterdam The Netherlands; ^4^ Center for Proteomics and Metabolomics LUMC Leiden The Netherlands; ^5^ Department of Molecular Hematology Sanquin Research Amsterdam The Netherlands; ^6^ Institute for Hygiene and Applied Immunology Center of Pathophysiology, Infectiology and Immunology Medical University of Vienna Wien Austria; ^7^ Swammerdam Institute for Life Sciences University of Amsterdam Amsterdam Netherlands

## Abstract

The development of an effective antitumor response relies on the synergistic actions of various immune cells that recognize tumor cells via distinct receptors. Tumors, however, often manipulate receptor–ligand interactions to evade recognition by the immune system. Recently, we highlighted the role of neolacto‐series glycosphingolipids (nsGSLs), produced by the enzyme β1,3‐*N*‐acetylglucosaminyltransferase 5 (B3GNT5), in tumor immune escape. We previously demonstrated that loss of signal peptide peptidase like 3 (SPPL3), an inhibitor of B3GNT5, results in elevated levels of nsGSLs and impairs CD8 T cell activation. The impact of loss of SPPL3 and an elevated nsGSL profile in tumor cells on innate immune recognition remains to be elucidated. This study investigates the antitumor efficacy of neutrophils, NK cells, and γδ T cells on tumor cells lacking SPPL3. Our findings demonstrate that SPPL3‐deficient target cells are less susceptible to trogocytosis by neutrophils and killing by NK cells and γδ T cells. Mechanistically, SPPL3 influences trogocytosis and γδ T cell‐instigated killing through modulation of nsGSL expression, whereas SPPL3‐mediated reduced killing by NK cells is nsGSL‐independent. The nsGSL‐dependent SPPL3 sensitivity depends on the proximity of surface receptor domains to the cell membrane and the affinity of receptor–ligand interactions as shown with various sets of defined antibodies. Thus, SPPL3 expression by tumor cells alters crosstalk with immune cells through the receptor–ligand interactome thereby driving escape not only from adaptive but also from innate immunity. These data underline the importance of investigating a potential synergism of GSL synthesis inhibitors with current immune cell‐activating immunotherapies.

## Graphical abstract

Tumor and immune cells interact through receptor–ligand interactions. B3GNT5 produces nsGSL on tumor cells which is regulated by SPPL3. When SPPL3 is absent, elevated nsGSL levels hinder receptor–ligand binding, leading to reduced immune cell activation.

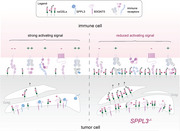

## 1 Introduction

To establish an effective antitumor response, immune cell receptors work synergistically to mediate and fine‐tune cellular responses. Functional interaction between receptors and their ligands is necessary to activate the right pro‐inflammatory immune cells or inhibit suppressive cells for a specific, yet balanced immune response. Well‐known is the human leukocyte antigen class I (HLA‐I) which presents (neo)antigens from an infected or mutated cell, which are recognized by cognate T cell receptors (TCRs) on CD8 T cells, instigating T cell activation and antitumor effector function upon interaction [1]. Unfortunately, pro‐inflammatory receptor–ligand interactions can be impeded or modulated by tumor cells to avoid proper recognition by the innate and adaptive immune system. Tumor evasion strategies have extensively been studied, and new tumor evasion strategies focusing on tumor‐specific traits are still being discovered.

Recently, we demonstrated an important role for signal peptide peptidase like 3 (SPPL3) in supporting adaptive immune responses through inhibition of the neolacto‐series glycosphingolipid (nsGSL) synthesis pathway. We identified that SPPL3, in addition to the previously described destruction of enzymes involved in *N*‐glycosylation of proteins, also destroys the enzyme β1,3‐*N*‐acetylglucosaminyltransferase 5 (B3GNT5), which is a key catalyst in the production of nsGSLs [2, 3, 4]. In general, GSLs are fundamental components of the cell membrane that regulate cell proliferation, differentiation, protein function, and intercellular receptor–ligand interactions in health but may also contribute to pathology [5, 6, 7, 8, 9, 10]. Our data showed that elevated B3GNT5‐produced nsGSL levels impair accessibility of HLA‐I leading to a reduced CD8 T cell activation in vitro [11]. In addition, we showed that antibody binding to HLA‐I domains proximal to the plasma membrane is relatively more affected by nsGSLs than to HLA‐I regions that were more distal to the plasma membrane. Moreover, the interactions of LIR‐1 and killer cell immunoglobulin‐like‐receptor‐2DL (KIR2DL2) fusion proteins with HLA‐I were impaired by nsGSLs, implying that nsGSLs can interfere with important immune receptor–ligand interactions.

Another study demonstrated that CD19 on SPPL3‐negative target cells was badly bound by antibodies leading to increased resistance to CD19 CAR T cell activity. In this case, SPPL3 directed this effect through control of *N*‐glycosylation of CD19 [12].

There are also studies that implicate that loss of SPPL3 may impair innate immune responses. One study showed that suppression of nsGSL synthesis through SPPL3 activity allows for better accessibility of the complement regulator CD59 [13]. Another study illustrated that genetic loss of SPPL3 led to a decreased susceptibility of B cell lymphoma cell line NALM6 to NK cell‐mediated cytotoxicity [14]. These findings were also observed by Zhuang et al. who, additionally, showed impaired binding of NK cell receptors NKG2D and CD2 to their partners MICA/B and CD58 on cells lacking SPPL3 [15]. This impaired binding was due to increased complex *N*‐glycans whose synthesis was no longer inhibited by SPPL3. Yet, whether the underlying mechanism of these observations also involves modulation of GSLs is not clear.

Together with our previous findings, this implies that the regulatory function of SPPL3 may control both adaptive and innate immune responses, possibly through both nsGSLs and *N*‐glycosylation. On a molecular level, this may be regulated through determining the accessibility of a potentially large variety of membrane proteins.

In this study, the nsGSL‐dependent and ‐independent role of regulation by SPPL3 in target cells on neutrophil, NK cell, and γδ T cell function was first explored. We show that target cells lacking SPPL3 are protected against trogocytosis by neutrophils and killing by NK cells and γδ T cells. Mechanistically, modulation of nsGSL expression by SPPL3 formed the basis for the SPPL3 sensitivity of trogocytosis by neutrophils or killing by γδ T cells, but not of killing by NK cells. We then determined SPPL3‐ and nsGSL‐sensitivity for many individual cell surface receptors and established that membrane receptor size and affinity between interacting proteins are crucial for nsGSL‐driven shielding. Interestingly, where most receptor–ligand/antibody interactions were SPPL3 sensitive through nsGSL modulation, some interactions were affected by SPPL3 in an nsGSL‐independent fashion. All together, these results show a new mode of regulation of innate immune responses. Upon decreased expression or loss of SPPL3 by tumor cells, the cumulative immune escape effect may therefore involve both adaptive and innate antitumor immunity.

## 2 Materials and Methods

All antibodies and membrane dyes used are listed in Tables S1 and S2.

### 2.1 Cell Isolation and Culture

#### 2.1.1 Cell Lines

HAP1 and K562 cell lines were cultured in IMDM (Gibco), and NALM6 cells in RPMI 1640 (Gibco) supplemented with 10% FCS (Serana) and 1% antibiotics (penicillin‐streptomycin, Invitrogen). HEK293T cells were cultured in DMEM supplemented with 10% FCS, 20 µg/mL gentamycin (Gibco), 1% l‐glutamine (Gibco), and 0.05 mM 2‐mercapto‐ethanol (βME (Sigma)), and NB4 cells (kindly provided by Dr. Hanke Matlung, Sanquin Research, Amsterdam, The Netherlands) were cultured in IMDM supplemented with 10% FCS, 1% l‐glutamine, and 1% antibiotics (penicillin‐streptomycin). NALM6 cells were cultured with UGCG inhibitor Eliglustat as previously described [16]. All cells were cultured at 37°C and 5% CO_2_.

#### 2.1.2 Isolation of γδ T Cells and NK From Peripheral Blood Mononucleated Cells (PBMCs)

Buffy coats were acquired from Sanquin Blood Supply, Amsterdam, The Netherlands. All donors were healthy adults and had provided written informed consent in accordance with the protocol of the local institutional review board, the Medical Ethics Committee of Sanquin Blood Supply, and conform to the principles of the Declaration of Helsinki. From these buffy coats, PBMCs were isolated using Lymphoprep (Axis‐Shield PoC AS, Scotland) density gradient. γδ T cells were next isolated using a direct γδ TCR‐targeting isolation method [17]. In short, PBMCs were incubated with a PE‐conjugated mouse antihuman Vδ2 TCR (Biolegend) for 30 min on ice, washed with PBS/0.1% BSA, and incubated with anti‐mouse IgG microbeads (Miltenyi) prior to positive MACS isolation according to manufacturer's protocol. The γδ T cells were further purified using FACS. NK cells were isolated from the PBMCs using the NK cell isolation kit from Miltenyi according to manufacturer's protocol. The cells were negatively sorted using an LS column (Miltenyi) followed by a second LD column to remove more NK‐negative cells to ensure optimal purity.

#### 2.1.3 Culture of Primary Cells

Purified γδ T cells were expanded for 14 days as previously described [17]. In short, the cells were cultured IMDM supplemented with 5% FCS, 5% human serum (HS, Sanquin), 1% antibiotics, 1% l‐glutamine with PHA (1 µg/mL, Remel Europe), IL‐2 (120 U/mL, Peprotech), IL‐7 (20 ng/mL, Miltenyi), and IL‐15 (20 ng/mL, Peprotech) together with feeder cells (irradiated PBMCs and Epstein–Barr virus‐ [EBV‐] immortalized lymphoblastoid cell lines [LCLs]). After expansion, the γδ T cells were immediately used for functional assays. Isolated NK cells were cultured O/N in RPMI 1640 with 100 ng/mL IL‐15 and used in functional assays the next day. Cells were cultured at 37°C and 5% CO_2_.

### 2.2 Genome Editing

A plasmid containing the gRNA‐targeting SPPL3 or UGCG (lentiCRISPR‐v2‐SPPL3gRNA‐puro [11]) was co‐transfected into HEK293T cells together with packaging plasmids psPAX2, pVSVg, and pAdVAntage (Promega) using Genejammer (Agilent) for virus production. Viral supernatant was filtered and used for transduction of NALM6 and K562 cells through spinoculation together with 8 µg/mL protamine sulfate (Merck). Transduced cells were selected using puromycin (1 µg/mL).

### 2.3 Functional Assays

#### 2.3.1 Coculture Assays With NK Cells

NALM6 or K562 target cell lines were plated two hours before coculture with NK cells in 50 µL complete RPMI (10% FCS and 1% antibiotics), at a density of 30,000 cells/well in a round‐bottom 96‐well plate. Next, NK cells were added to the NALM6 cells at an effector‐to‐target (E:T) ratio of 10:1, 5:1, 2.5:1, 1:1, and 0.5:1 or to K562 cells at a 5:1, 1:1, and 0:1 ratio in a total volume of 100 µL complete RPMI. After a 5‐h coculture, cells were harvested and transferred to a V‐bottom plate, after which the percentage of dead target cells was determined using flow cytometry. NK cells were distinguished using anti‐CD16 (Biolegend) and anti‐CD56 (BD Horizon). The percentage of dead target cells observed in wells without NK cells was subtracted from the percentage of dead target cells in wells with NK cells to determine the percentage of target cell death due to coculture with NK cells.

#### 2.3.2 Coculture Assays With γδ T Cells

HAP1 target cell lines were plated 1 day prior to the coculture with γδ T cells at a density of 15,000 cells/well in a flat‐bottom 96‐well plate and treated O/N or not with 10 µM pamidronate (PAM, Sigma) in complete IMDM (includes 10% FCS, 1% antibiotics). After the O/N culture, the wells were carefully washed to remove dead cells, and γδ T cells were added at a 1:1, 5:1, and 10:1 ratio (E:T) in a total volume of 100 µL of complete IMDM. After a 5‐h coculture, the cells were harvested and transferred to V‐bottom plates. Target cells that remained attached after initial harvest were detached using trypsin followed by PBS, added to the V‐bottom plate, and washed altogether. The percentage of dead target cells and the magnitude of γδ T cell activation were measured using flow cytometry. The γδ T cells were identified with anti‐CD3 (BD Horizon) and anti‐Vδ2 TCR (Biolegend), which is the same antibody used to sort the γδ T cells prior to expansion and analyzed for CD25, CD69, and granzyme B expression. The percentage of dead target cells, gated on the γδ T cell negative fraction, observed in wells without γδ T cells was subtracted from the percentage of dead target cells in wells with γδ T cells to determine the percentage of target cell death due to coculture with γδ T cells.

#### 2.3.3 Trogocytosis Assays

Prior to the assay, 0.5·10^6^ NB4 cells/mL were differentiated with 5 µM all‐*trans* retinoic acid (ATRA; Sigma‐Aldrich) for 7 days to promote expression of the dimer CD11b/CD18 (MAC‐1) which is required for trogocytosis [18, 19]. As a readout for proper maturation, expression of CD11b on stimulated NB4 cells was determined by flow cytometry. Next, NB4 cells were labeled with 2.5 µM violet proliferation dye 450 (VPD450, BD) for 30 min at RT. The HAP1 cells were labeled with 5 µM DiO lipophilic membrane dye (Invitrogen) for 30 min at RT. After labeling and washing, HAP1 cells were incubated together with NB4 cells at 37°C in a 96‐well U‐bottom plate at a 5:1 E:T ratio up to 4 h. Samples were fixed with stop‐buffer containing 0.5% PFA and 1% BSA in PBS after 1, 2, and 4 h. The percentage of NB4 cells positive for DiO was assessed by flow cytometry.

### 2.4 Flow Cytometry

All antibodies and membrane dyes used are listed in Tables S1 and S2.

Prior to staining, cells were washed with PBS/0.1% BSA. Extracellular staining was performed through incubation with specific antibodies and LIVE/DEAD NEAR‐IR (Invitrogen) diluted in PBS for 30 min on ice in the dark. Next, cells were washed twice with PBS/0.1% BSA and either resuspended in PBS and directly analyzed or fixated using the BD Cytofix/Cytoperm fixation and permeabilization kit (BD Bioscience) according to the manufacturer's protocol. For intracellular staining, cells were permeabilized and incubated with antibodies diluted in permeabilization buffer for 30 min on ice in the dark. Subsequently, cells were washed twice and resuspended in PBS/0.1% BSA prior to analysis. Stained cells were analyzed on BD flow cytometers (LSR‐II, Fortessa, FACSymphony) or sorted (ARIA‐II) and analyzed using FlowJo software version 10.9.0 (Ashland, OR: Becton, Dickinson and Company; 2023).

#### 2.4.1 Protein Panel Methods, Quality Control, and Analysis

Prior to antibody staining, HAP1 WT cells were labeled with cell trace violet (Thermo Fisher Scientific), HAP1 SPPL3^−/−^ cells with AF350 succinimidyl ester (Invitrogen), and HAP1 SPPL3^−/−^B3GNT5^−/−^ with both according to manufacturer's protocol [20]. HAP1 B3GNT5^−/−^ cells remained unlabeled. Cells were washed thoroughly and mixed in a 1:1:1:1 ratio. Subsequently, the mixture of cells was incubated with different dilutions of 168 unique, primary antibodies which come from the Human Leukocyte Differentiation Antigen Typing Workshops, commercial suppliers, or are in‐house produced (listed in Table S2), targeting 34 cell surface proteins for 30 min on ice [21, 22, 23]. Cells were washed twice followed by staining with anti‐mouse IgG‐AF647 (Invitrogen) for 30 min on ice in the dark. The cells were washed and resuspended in PBS/0.1% BSA for analysis.

Antibodies were excluded from analysis if there was no positive stain compared to the secondary antibody‐only control. Only proteins of which it was established that their cell surface expression itself was not altered in one of the cell lines, as established by using saturating concentrations of at least one antibody clone per target protein. To determine whether epitope accessibility was affected by nsGSLs, the mean fluorescence intensity (MFI) for each antibody at a sub‐saturating concentration was compared among the different cell lines. Epitopes were considered to be affected by the loss of SPPL3 if antibody binding to HAP1 SPPL3^−/−^ cells was different compared to WT at non‐saturating antibody concentrations (MFI SPPL3^−/−^/MFI WT <0.8 or >1.2) and by nsGSLs if (additional) knockout of B3GNT5 showed antibody binding comparable to or higher than WT levels (MFI B3GNT5^−/−^/MFI WT >0.8 or MFI SPPL3^−/−^B3GNT5^−/−^/MFI WT >0.8).

#### 2.4.2 Flow Cytometry Analysis of the CD147‐Targeting Antibody Panel

HAP1 WT cells were labeled with AF350 succinimidyl ester, HAP1 SPPL3^−/−^ cells with cell trace violet, and HAP1 SPPL3^−/−^B3GNT5^−/−^ with CFSE (Invitrogen) according to manufacturer's protocol [20]. HAP1 B3GNT5^−/−^ cells remained unlabeled. Cells were washed twice with PBS/0.1% BSA, mixed in a 1:1:1:1 ratio, and incubated with LIVE/DEAD NEAR‐IR for 30 min on ice. Cells were washed twice and incubated with 13 different anti‐CD147‐targeting antibodies in 50 µL PBS [24], at concentrations of 20, 1, or 0.05 µg/mL and incubated for 30 min on ice. Hereafter, cells were washed twice and resuspended in 35 µL goat anti‐mouse IgG (Invitrogen) in PBS for 30 min on ice. Once more, cells were washed twice and resuspended in PBS/0.1% BSA prior to analysis using flow cytometry. Non‐saturating antibody concentrations were selected for analysis. The effect of SPPL3^−/−^ on the binding of the antibodies was presented as the ratio MFI SPPL3^−/−^/MFI WT.

#### 2.4.3 Flow Cytometry Using Fusion Proteins

Fusion proteins LIR1‐Fc, NKG2D‐Fc, SIRPα‐Fc, and Siglec7‐Fc (R&D Biosystems) were reconstituted in PBS (250 µg/mL) prior to use. HAP1 cell lines were trypsinized and washed with PBS/0.1% BSA, and WT cells were labeled with DIL membrane dye (Invitrogen), HAP1 SPPL3^−/−^ cells with VPD450 membrane dye (BD), and HAP1 SPPL3^−/−^B3GNT5^−/−^ with CFSE according to manufacturer's protocol [20]. Next, the cells were incubated with LIVE/DEAD NEAR‐IR diluted in PBS for 30 min on ice in the dark. The cells were washed twice with PBS/0.1% BSA and mixed in a 1:1:1:1 ratio. Hereafter, cells were resuspended in 40–48 µL of a dilution series of the fusion protein solution (starting at 12 or 10 µg, twofold dilutions) for 60 min at RT protected from light. Cells were washed twice to remove unbound proteins and incubated with APC antihuman IgG (Biolegend) for 30 min on ice and protected from light. After incubation, cells were washed with PBS/0.1% BSA and resuspended in PBS/0.1% BSA prior to analysis.

### 2.5 In Silico Protein Height Measurement

The maximal distance between amino acids of the extracellular domain (ECD) of the cell surface proteins and the first amino acid after the transmembrane region was modeled using PyMOL (Schrödinger LLC, version 2.5.8) with protein structures imported from the Protein Data Bank (PDB) modeled with AlphaFold. The distance in Ångström (Å) between the alpha‐carbons of all possible amino acid residue pairs and the first amino acid after the transmembrane region was calculated, and the longest distance was selected from this matrix. For the integrins CD49b, CD49e, and CD51, the inactive conformation was used to model maximal distance.

### 2.6 Mass Spectrometry: Total Protein Abundances

HAP1 WT and SPPL3^−/−^ were prepared and analyzed as described previously [25].

The raw mass spectrometry acquisition files and MaxQuant search files have been deposited to the ProteomeXchange Consortium via the PRIDE partner repository with the dataset identifier PXD056501.

### 2.7 Mass Spectrometry: Glycosphingolipid‐Derived Glycan Analyses

NALM6 WT and SPPL3^−/−^ as well as K562 WT, UGCG^−/−^, and SPPL3^−/−^ were prepared for‐ and analyzed with porous graphitized carbon LC‐ESI‐MS/MS (PGC LC–MS) as described previously [11].

### 2.8 Statistical Analysis

Statistical testing was done by a Student's *T*‐test or a one‐way ANOVA followed by a Tukey's multiple comparison test. The statistical analysis was performed using GraphPad Prism version 10.0 for Mac OS (GraphPad Software, Boston, Massachusetts, USA). Differences were considered significant when *p* ≤ 0.05.

## 3 Results

### 3.1 SPPL3 Supports Antitumor Cell Cytotoxicity of γδ T Cells

**FIGURE 1:**
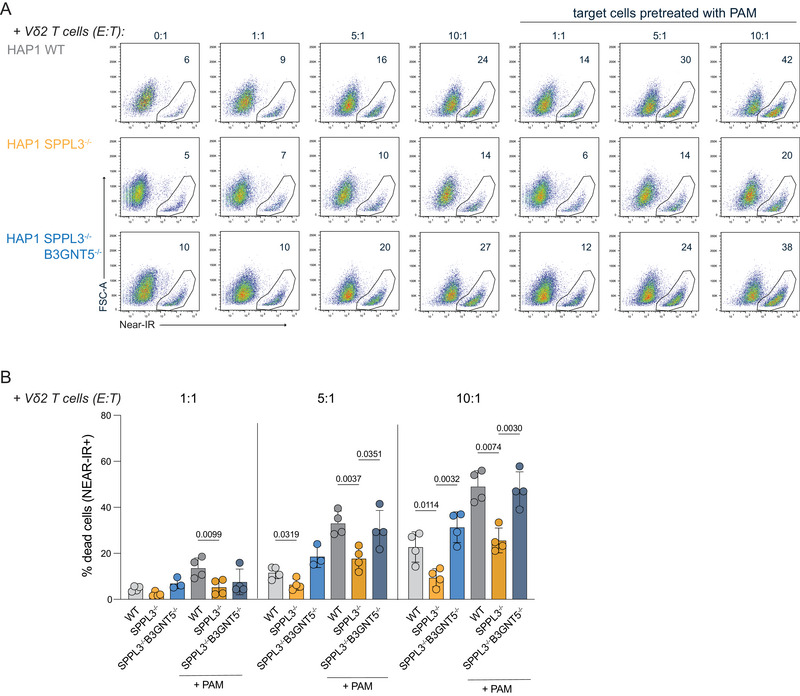
nsGSLs impair antitumor responses of γδ T cells. Freshly expanded γδ T cells from four donors were incubated with HAP1 cells (WT, SPPL3^−/−^, and SPPL3^−/−^B3GNT5^−/−^) for 5 h in a 1:1, 5:1, and 10:1 effector‐to‐target (E:T) ratio. The target cells were either untreated or pretreated with PAM O/N prior to culture. Triplicates were used for each donor. Representative flow cytometry plots (A) and combined data of *n* = 4 (B) showing the percentage of dead target cells (near‐IR positive) without and with addition of γδ T cells in different ratios. Data shown are representative from four experiments with four donors per experiment and three technical replicates per datapoint. A one‐way ANOVA was used to assess statistical significances. PAM, pamidronate.

Previously, we demonstrated that the loss of SPPL3 in tumor target cells reduced effector cytokine production and tumor cell clearance by αβ T cells due to nsGSL‐mediated shielding of HLA‐I [11]. To assess whether SPPL3 affects the target cell recognition and anti‐target cell cytotoxicity of γδ T cells, γδ T cells were expanded from PBMCs and cocultured with target HAP1 cells. Killing of HAP1 SPPL3^−/−^ cells by γδ T cells was reduced (Figure 1A,B). This SPPL3‐dependent reduction in cytotoxicity was alleviated in the absence of the nsGSL‐producing enzyme B3GNT5 (SPPL3^−/−^B3GNT5^−/−^) (Figure 1A,B), demonstrating that the insensitivity to γδ T cells cytotoxicity of SPPL3^−/−^ target cells involves nsGSLs. To stimulate tumor cell killing, target cells were in some cases pretreated with PAM [17]. PAM pretreatment of the WT and SPPL3^−/−^ target cells made both cell types more susceptible to killing by γδ T cells, but the reduced cytotoxicity of γδ T cells against SPPL3^−/−^ compared to WT target cells remained (Figure 1A,B). A lower expression of the activation marker CD25 was observed on the γδ T cells after coculture with SPPL3^−/−^ cells compared to WT or SPPL3^−/−^B3GNT5^−/−^ cells (Figure S1A,B). In contrast, their expression of CD69 was elevated after incubation with SPPL3^−/−^ target cells compared to the other target cells (Figure S1A,D,E). Granzyme B levels remained unchanged (Figure S1A,C).

Together, the data show that the loss of SPPL3 and subsequent upregulation of nsGSLs by tumor cells protect them from elimination by γδ T cells and promote CD69 upregulation by γδ T cells.

### 3.2 SPPL3 Sensitizes Tumor Cells for NK Cell‐Mediated Lysis Through an nsGSL‐Independent Pathway

The link between regulation of *N*‐glycosylation by SPPL3 and decreased sensitivity of tumor cells to NK cell activity has been described [15]. To assess whether, similar to γδ T cells, NK‐mediated killing can be influenced by loss of SPPL3 in an nsGSL‐sensitive fashion, the killing effectivity of K562 SPPL3^−/−^ and NALM6 SPPL3^−/−^ target cells by NK cells and a potential involvement of nsGSLs was investigated.

**FIGURE 2:**
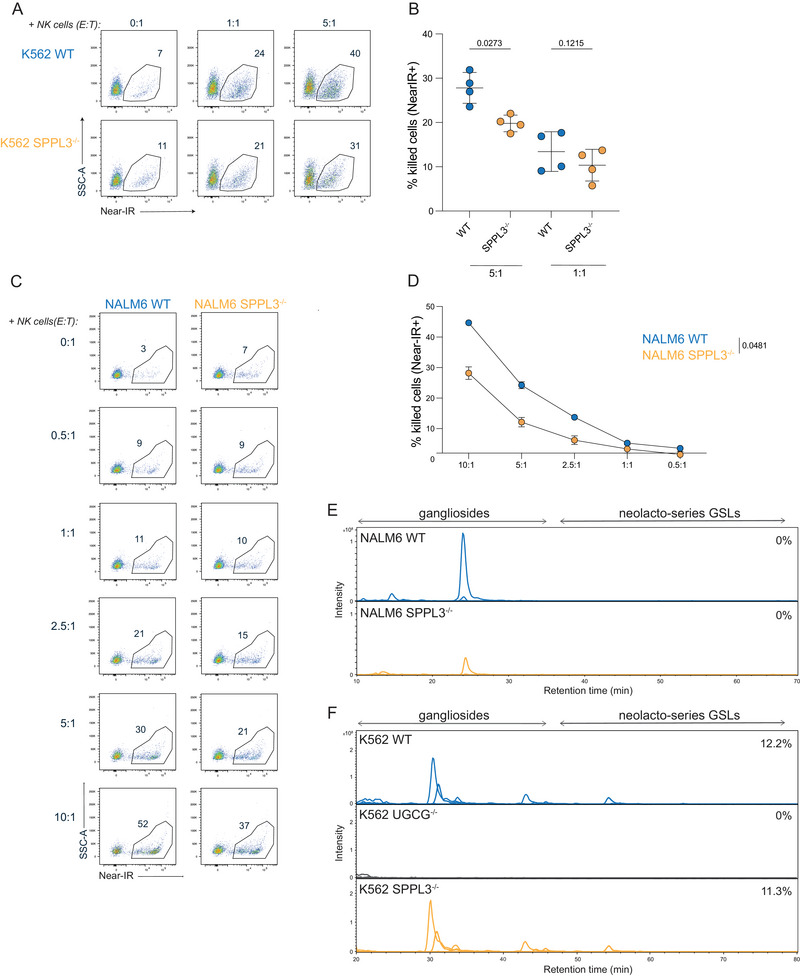
SPPL3 regulates NK cell immune responses independent of nsGSLs. NK cells were cocultured with NALM6 or K562 target cells for 5 h in different E:T ratios. (A) Representative flow cytometry plots and (B) combined data illustrating the proportion of dead K562 WT and SPPL3^−/−^ cells (near‐IR positive) cocultured with NK cells in a 0:1, 1:1, and 5:1 ratio. Data shown are representative from three experiments with four donors per experiment and three technical replicates per datapoint. Representative flow cytometry plots (C) and combined data of *n* = 2 (D) showing the percentage of dead (near‐IR positive) NALM6 WT or SPPL3^−/−^ cells with different E:T ratios. Data shown are representative from three experiments with two donors per experiment and three technical replicates per datapoint. (E and F) Extracted ion chromatograms of total GSL glycan content released from (E) NALM6 WT and SPPL3^−/−^ cells as well as (F) K562 WT, UGCG^−/−^, and SPPL3^−/−^ cells using PGC LC–MS. A paired Student's *T*‐test (B) or a one‐way ANOVA (D) was used to assess statistical significances.

Coculture of isolated primary NK cells with WT or SPPL3^−/−^ K562 cells at different E:T ratios demonstrated a superior killing activity against WT compared to SPPL3^−/−^ cells (Figure 2A,B), in line with the previous report. Similar results were observed for NK cocultures with WT and SPPL3^−/−^ NALM6 cells (Figure 2C,D). To investigate whether the observed effect was due to a difference in the GSL repertoire, the glycan profiles of K562 and NALM6‐derived GSLs were analyzed for nsGSL expression using PGC LC‐MS. K562 UGCG^−/−^ cells were taken along as a control as UGCG is a key enzyme required for GSL production [11]. Loss of SPPL3 did not promote upregulation of nsGSLs in these cells (Figure 2D,E). In addition, NALM6 WT and SPPL3^−/−^ cells were treated with the GSL synthesis inhibitor Eliglustat and stained with the antibody W6/32‐targeting HLA‐I which was previously shown to be inhibited by nsGSLs on HAP1 cells. Control SPPL3^−/−^ HAP1 cells recapitulated the loss of staining after Eliglustat treatment, but staining of SPPL3^−/−^ NALM6 cells remained lower compared to WT NALM6 cells (Figure S2) [11].

These data together showed that killing of tumor cells by NK cells is inhibited by the loss of SPPL3, but this SPPL3‐mediated effect is likely not caused by elevated nsGSLs on the cell surface.

### 3.3 Trogocytosis of SPPL3^−/−^ Cells by Neutrophil‐Like NB4 Cells Is Diminished

**FIGURE 3:**
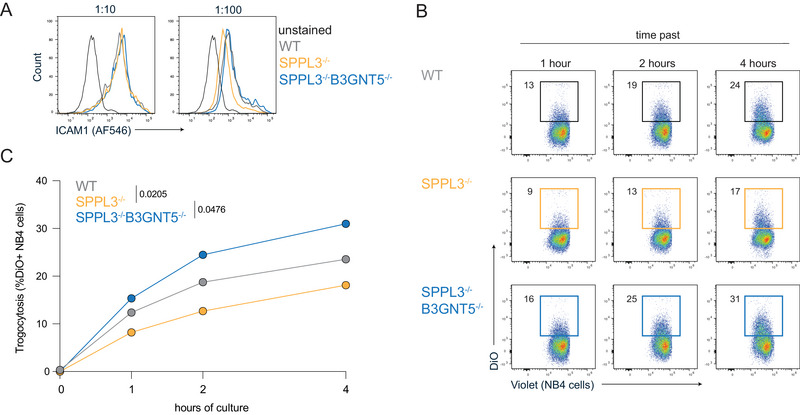
Trogocytosis of HAP1 SPPL3^−/−^ cells is diminished due to nsGSLs. (A) Histogram illustrating ICAM1 staining of HAP1 WT, SPPL3^−/−^, and SPPL3^−/−^B3GNT5^−/−^ cells with a saturating antibody dilution (1:10, left plot) and non‐saturating dilution (1:100, right plot). The data represent two independent experiments with three technical replicates. Trogocytosis of HAP1 cells (stained with a DiO dye) was determined by the gain of the DiO dye by NB4 cells (stained with a violet membrane dye) over a time. (B) Representative flow cytometry plots and combined data (C) representing four independent experiments with three technical replicates per datapoint. A paired one‐way ANOVA was used to assess statistical significances.

Next, we investigated whether the loss of SPPL3 in target cells has an effect on neutrophil functionality, another innate immune cell type that is described to mediate antitumor reactivity through trogocytosis of cell membrane fragments from tumor cells [18]. This process involves multiple protein–protein interactions that are potentially influenced by SPPL3‐regulated nsGSL levels. An example of such interaction is the binding of the CD11b/CD18 (MAC‐1) dimer on neutrophils to ICAM1 expressed by target cells. Therefore, we evaluated the effect of SPPL3 deletion on antibody binding to ICAM1 on HAP1 target cells. With saturating antibody concentrations, there was no difference observed in ICAM1 staining between WT, SPPL3^−/−^, or SPPL3^−/−^/B3GNT5^−/−^ cells, indicating that ICAM1 protein expression is the same on all cells (Figure 3A, left plot). On the contrary, under non‐saturating conditions, SPPL3^−/−^ cells showed moderately lower cell surface staining for ICAM1 compared to WT cells, which was alleviated by the additional depletion of nsGSLs (Figure 3A, right plot). To investigate whether differences in protein accessibility affected neutrophil function, we chose to analyze trogocytosis. Therefore, neutrophil‐like NB4 cells were first stimulated with ATRA for 7 days to promote CD11b/CD18 expression and maturation (Figure S3). Evaluation of trogocytosis after ensuing coculture with HAP1 cells, as measured through HAP1‐derived DiO‐label acquisition by NB4 cells over time, showed that the efficiency of SPPL3^−/−^ HAP1 cells was lower compared to trogocytosis of WT cells (Figure 3B,C). Additional deletion of B3GNT5 improved the trogocytosis efficiency of HAP1 SPPL3^−/−^ cells. Together, these data show that SPPL3 can affect target cell trogocytosis by neutrophils, which is mediated by nsGSLs (Figure 3B,C).

### 3.4 Loss of SPPL3 Affects Receptor–Ligand Interactions Through nsGSL‐Dependent and ‐Independent Mechanisms

Given that nsGSL‐mediated inhibition of interactions is dependent on its affinity, we then investigated to which extent nsGSLs influence receptor–ligand interactions which are generally lower affinity than antibody‐protein interactions. Therefore, fusion proteins of a panel of different immune receptors were used that consist of an Fc tail fused to the ECD. We used fusion proteins of the phagocytosis inhibitory receptor SIRPα (ligand: CD47), expressed by myeloid cells such as neutrophils and macrophages, and Siglec‐7 which is a sialic acid‐binding inhibitory receptor mainly expressed on NK cells and to a lower extent on CD8 T cells and monocytes, and the activating receptor NKG2D (ligand: MICA/B, ULBPs), expressed by NK, NKT, γδ T cells, and activated macrophages [26, 27].

**FIGURE 4:**
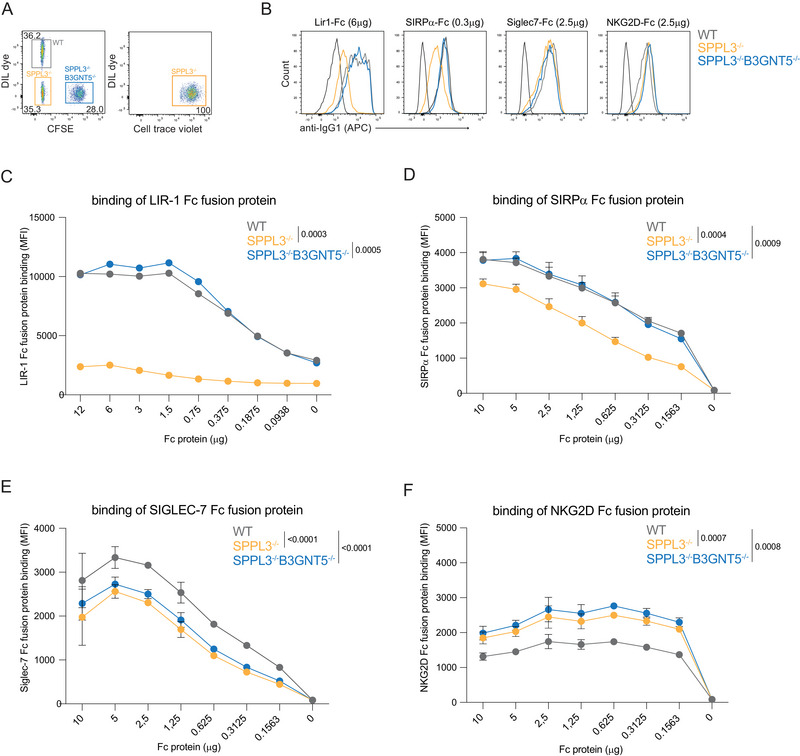
SPPL3 regulates Fc‐protein binding to their ligands on HAP1 WT, SPPL3^−/−^, and SPPL3^−/−^B3GNT5^−/−^ cells. HAP1 WT (DiL dye), SPPL3^−/−^ (Celltrace violet), and SPPL3^−/−^B3GNT5^−/−^ (CFSE) were mixed (A) and incubated with different concentrations of LIR1‐Fc, SIRPα‐Fc, Siglec‐7‐Fc, or NKG2D‐Fc. Representative flow cytometry plots (B) and summarizing graphs for the binding of LIR‐1‐Fc (C), SIRPa‐Fc (D), Siglec‐7‐Fc (E), and NKG2D‐Fc (F). Data represent two (Siglec‐7‐fc and NKG2D‐fc) or three (LIR‐1‐fc and SIRPα‐fc) with two technical replicates per datapoint. A paired one‐way ANOVA was used to assess statistical significances. MFI, mean fluorescence intensity.

Previously, binding of a leukocyte immunoglobulin‐like receptor B1 (LIR1, LILRB1) fusion protein to HLA‐I was shown to be highly impaired by nsGSLs [11]. To validate that the method used here allows for identification of differential binding in the presence of many nsGSLs, the impaired binding of LIR1‐Fc to HAP1 SPPL3^−/−^ was confirmed (Figure 4A–C).

The interaction of the SIRPα‐Fc with CD47 was impaired after loss of SPPL3. This could be restored by deletion of B3GNT5, indicating that nsGSLs impaired binding of SIRPα (Figure 4B,D). The binding of Siglec‐7 to SPPL3^−/−^ cells was reduced to some degree, but independently of nsGSLs produced by B3GNT5 (Figure 4B,E). Likewise, the interaction between NKG2D and probable binding partners MICA/B and/or ULPBs was moderately improved with the loss of SPPL3, independent of B3GNT5‐produced nsGSLs (Figure 4B,F).

Altogether, multiple receptor–ligand interactions are modulated depending on SPPL3 expression through nsGSL‐dependent and ‐independent mechanisms.

### 3.5 The Accessibility of Several Membrane Proteins Is Affected by nsGSLs

We previously demonstrated that enhanced nsGSLs in SPPL3^−/−^ cells affect the accessibility of several epitopes on HLA‐I [11]. To assess whether the effect of SPPL3 and nsGSL expression in target cells on NK cells, γδ T cells, and neutrophils is driven by the cumulative effect of different affected cell surface proteins, we assessed the effect of SPPL3 and nsGSL expression on 34 different cell surface proteins using a panel of 168 antibodies. First, we employed a proteomics approach to exclude different protein expressions of the targeted proteins which could bias analysis of antibody binding (Figure S5A,B). Using mass spectrometry, we found that only one protein (glutathione *S*‐transferase mu 3 [GSTM3]) was significantly differentially expressed between HAP1 WT and SPPL3^−/−^ cell lines. Thus, for none of the proteins used in our antibody panel, there was a significant difference in protein expression detected (Figure S5C).

**FIGURE 5:**
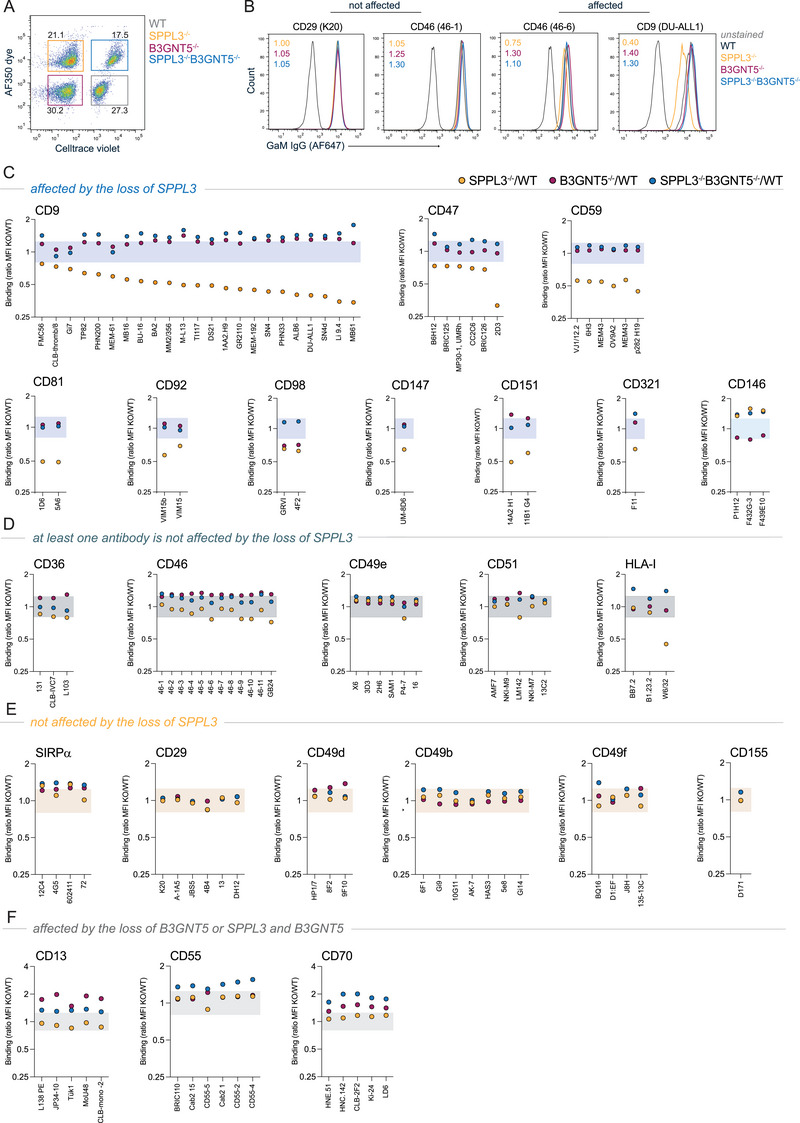
Antibody binding to proteins on HAP1 SPPL3^−/−^ and HAP1 SPPL3^−/−^B3GNT5^−/−^ relative to HAP1 WT cells. HAP1 WT (Celltrace violet), SPPL3^−/−^ (AF350 dye) and SPPL3^−/−^B3GNT5^−/−^ (Celltrace violet and AF350 dye) (A) were mixed and incubated with 168 unique, primary antibodies targeting 34 cell surface proteins. Antibodies targeting a total of 24 proteins passed quality control. (B) A proportion of proteins were targeted with antibodies that were all not affected to bind to their epitopes with SPPL3^−/−^, whereas some proteins were partially (at least one of the tested antibodies) or completely affected (all tested antibodies), which could be alleviated with additional B3GNT5^−/−^. The ratio of the MFI of each antibody for the SPPL3^−/−^, B3GNT5^−/−^, and SPPL3^−/−^B3GNT5^−/−^ compared to the WT cells was calculated (example in plots in B). The proteins were grouped on the basis of the effect of the loss of SPPL3 or B3GNT5; proteins with epitopes affected by the loss of SPPL3 (C), proteins of which at least one antibody is not affected by the loss of SPPL3 (D), proteins with epitopes that are not affected by the loss of SPPL3 (E), proteins with epitopes affected by the loss of B3GNT5 or SPPL3 and B3GNT5 (F). The colored bar represents the area that is considered similar to WT (cutoff for difference: >0.8 and <1.2). Data represent two independent experiments and three technical replicates per datapoint. MFI, mean fluorescence intensity.

Next, the various HAP1 cell lines were barcoded with different membrane dyes and mixed prior to antibody staining to allow for the most optimal comparison (Figure 5A). Out of the 34 proteins assessed with this panel, the antibody staining of 24 proteins passed quality control (detailed requirements are listed in the methods, listed in Table S2). To exclude possible differences of protein expression between cell lines, antibodies showing differential staining of the cell lines under saturating conditions were excluded. Per antibody, the ratio of the MFI between each KO cell line and the WT cell line was determined. Ratios between 0.8 and 1.25 were considered similar to WT (examples in Figure 5B, left panel), and values below or above this cutoff were considered different (examples in Figure 5B, right panel).

On the basis of this analysis, we could discriminate between proteins that were affected, not affected, or at least in part not affected by loss of SPPL3 (Figure 5B–F). In contrast to the other proteins, accessibility of CD146 by its specific antibodies was increased in the absence of SPPL3 (Figure 5C). The group of proteins for which all antibodies tested were negatively affected by SPPL3 KO consisted of CD9, CD47, CD59, CD81, CD92, CD98, CD147, CD151, and CD321 (Figure 5C). SPPL3 effects could be reversed by additional depletion of nsGSLs, demonstrating that SPPL3 loss affected these proteins through modulation of nsGSLs.

At least one, but not all, antibodies targeting the proteins CD36, CD46, CD49e, and CD51 were not affected in the HAP1 SPPL3^−/−^ cells, similar to antibodies targeting HLA‐I as described (Figure 5B,D) [11]. Antibody binding to SIRPα, CD29, CD49b, CD49d, CD49f, and CD155 was generally equal between the tested cell lines (Figure 5B,E). A small subgroup of proteins was specifically affected by the loss of B3GNT5, but not SPPL3 (Figure 5F).

### 3.6 nsGSLs Do Not Limit Accessibility to Proteins With Long ECDs

We hypothesized that antibody epitopes more distal from the cell membrane may not be subjected to shielding by nsGSLs. However, for most of the antibodies used, the exact epitopes have not been properly mapped. Therefore, we investigated whether the size of the ECD correlated with the nsGSL‐dependent SPPL3 sensitivity of those proteins.

**FIGURE 6:**
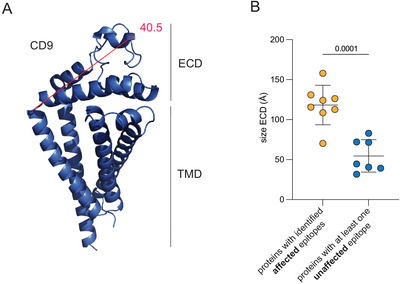
The protrusion of proteins affected by nsGSLs versus unaffected proteins. Available protein structures were obtained from the Alphafold database for the proteins that contained epitopes that were affected (Figure 5C,D) or not affected by nsGSLs (Figure 5E). The distance between the alpha carbons of the first amino acid of the extracellular domain to all other amino acids was determined in Armstrong (Å) using PyMOL, and the longest theoretical distance was selected. For example, (A) the maximum distance of CD9 is 40.5 Å. The rest of the structures are shown in Figure S4. (B) The maximum protruding distance (size) of proteins with versus the proteins without identified affected epitopes. ECD, extracellular domain.

For 15 proteins shown in Figure 5C–E, the structure was modeled in AlphaFold. The distance between the alpha carbons of each amino acid within the ECD and the alpha carbon of the ECD was determined, and the longest distance (Å) was used as the size of the ECD (Figure S4, Table 1). For example, the largest ECD of CD9 has a diameter of 40.5 Å (Figure 6A, Figure S4, Table 1).

**TABLE 1:** List of affected proteins and proteins with at least one non‐affected epitope.

Affected proteins	Size ECD (Å)	Proteins with at least one non‐affected epitope	Size ECD (Å)
CD9	40.5	CD46	112.8
CD47	44.6	CD49b	157.9
CD59	31.7	CD49d	108.5
CD81	39.2	CD49e	126.3
CD98	72.0	CD51	130.9
CD147	71.5	CD155	115.6
CD321	82.9	SIRPα	124.1
		HLA‐I	70.2

Abbreviations: ECD, extracellular domain; HLA‐I, human leukocyte antigen class I.

The ECD sizes of cell surface proteins that contain domains that are not affected by nsGSLs were significantly larger (µ = 126 ± 22 Å) than proteins for which we did not identify such domains (µ = 55 ± 20 Å) (Figure 6B). These data indicate that the accessibility of cell membrane proteins that extend less from the plasma membrane is more prone to be affected by loss of SPPL3 compared to proteins that extend further from the plasma membrane.

### 3.7 Severity of nsGSL Effect Is Dependent on Affinity of a Protein Interaction

**FIGURE 7:**
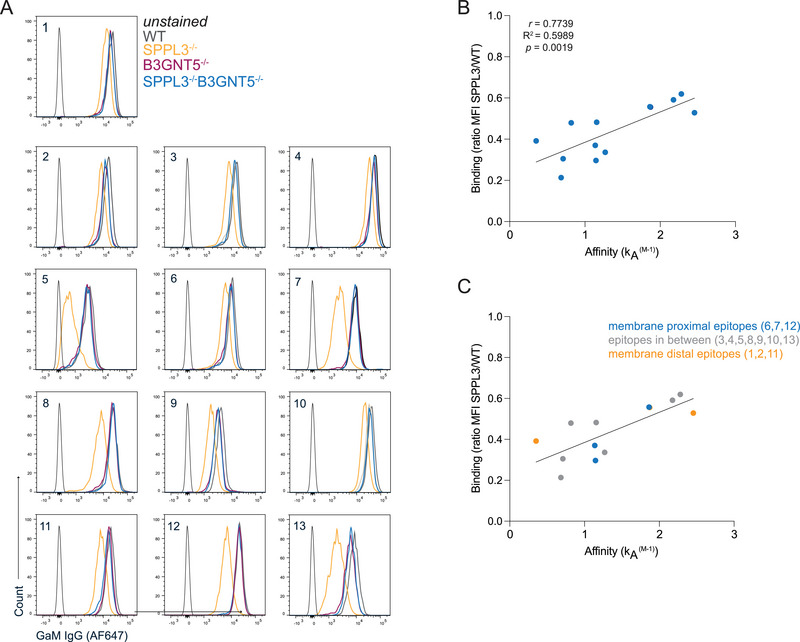
The binding of multiple anti‐CD147 antibodies with different affinities to HAP1 WT, HAP1 SPPL3^−/−^, HAP1 B3GNT5^−/−^, and HAP1 SPPL3^−/−^/B3GNT5^−/−^ cells. Thirteen different antibodies targeting CD147 were incubated with “barcoded” and mixed HAP1 WT (AF350 dye), SPPL3^−/−^ (Celltrace violet), B3GNT5^−/−^ (unstained), and SPPL3^−/−^/B3GNT5^−/−^ (CFSE) cells. (A) Representative flow cytometry plots for each antibody. (B) The correlation plot of the degree of SPPL3 sensitivity of binding for each antibody (SPPL3^−/−^ MFI/WT MFI) versus the affinity, as described by Koch et al., of the antibodies for their epitope 24. (C) Categorization of the epitopes targeted by the antibodies based on the position of the epitopes relative to the cell membrane. Data shown are representative for two independent experiments with three technical replicates per datapoint. MFI, mean fluorescence intensity.

We then wondered whether within the group of nsGSL‐affected interactions, affinity or further distance measures of such protein–protein interaction may play a role in the degree by which nsGSLs diminish binding. To test these hypotheses, we used CD147 as model antigen, as the previously tested antibody was affected by nsGSLs (Figure 5), as it has a significant ECD size of 71.5 Å (Table 1, Figure S4), and as we had access to 13 different CD147‐specific antibodies, each with well‐defined epitope positions and binding affinities as described by Koch et al. [24]. We then evaluated the loss of binding of these antibodies by nsGSLs by staining HAP1 cells with the different nsGSL profiles (WT, SPPL3^−/−^, B3GNT5^−/−^, and SPPL3^−/−^B3GNT5^−/−^). In comparison to WT cells, all 13 antibodies showed impaired binding to SPPL3^−/−^ cells (Figure 7A, Figure S5). The impaired binding of these antibodies was alleviated by additional deletion of B3GNT5, validating that nsGSLs were the cause of reduced binding to CD147 (Figure 7A). The respective antibody binding affinity correlated strongly with the degree of compromised binding (Figure 7B).

Because ECD size relates to the accessibility of an epitope (Figure 6), we assessed whether the position of the epitopes targeted by the CD147 antibodies in relation to the cell membrane would also play a role in the diminished binding. In their study, Koch et al. used a cross‐blocking analysis to predict the positions of the epitopes for each of the CD147‐targeting antibodies in relation to each other and the cell membrane [24]. Antibodies 6, 7, and 12 target membrane proximal epitopes, 1, 2, and 11 target membrane distal epitopes, and the others target epitopes in‐between. However, within this group of nsGSL‐affected antibodies, their epitope positions were not related to the severity of diminished binding (Figure 7C). Together, these data suggest that the degrees by which protein interactions with receptor domains are shielded by nsGSLs are additionally dictated by the affinity of the interaction.

## 4 Discussion

We showed that tumor cells that lack SPPL3 possess the ability to evade innate immune responses mediated by NK cells, γδ T cells, and neutrophils. The diminished responses of the latter two are driven by increased nsGSL levels. For both nsGSL‐dependent and ‐independent processes, the net cellular effect is likely caused by modulation of multiple receptor–ligand interactions in the absence of SPPL3. With a representative panel, we showed that several membrane proteins are affected by the loss of SPPL3 in an nsGSL‐sensitive fashion. Moreover, we established that the degree of nsGSL‐dependent and SPPL3 sensitivity of these proteins may be correlated to the size of surface receptors to the plasma membrane and the affinity between protein–protein interactions. Therefore, expression of SPPL3 by tumor cells influences crosstalk between immune cells through a multitude of receptor–ligand interactions thereby driving escape not only from adaptive but also from innate immunity.

In line with our data, Dufva et al. identified SPPL3 as a gene that facilitates susceptibility to NK cell‐mediated killing [28]. We showed that K562 and NALM6 SPPL3^−/−^ cells were more resistant to NK cell‐mediated killing without involvement of nsGSLs. However, the target cell lines utilized in this study did not show upregulation of nsGSLs with the loss of SPPL3. Therefore, we cannot use these targets to investigate whether nsGSLs influence NK cell‐mediated killing, which should be tested using target cell lines in which high nsGSL expression is established or engineered. Altered *N*‐glycosylation of ligands on tumor cells may be important for recognition by NK cells and may explain the lower NK cell‐mediated cell death of SPPL3^−/−^ compared to WT NALM6 cells presented in this work. Heard et al. showed that binding of anti‐CD19 antibodies to NALM6 SPPL3^−/−^ was impaired and, in addition, that the loss of SPPL3 in NALM6 cells increases their resistance towards CD19‐targeting CAR T cells [12]. Absence of SPPL3 results in increased and altered *N*‐glycosylation of CD19 which disrupts the CAR‐binding epitope. Both antibody binding and CAR T cell killing could be restored by using kifunensine, a potent inhibitor of mannosidase I, indicating an important role of *N*‐glycosylation in the recognition of target proteins in NALM6 cells. Zhuang et al. further identified multiple glycosyltransferases such as B3GNT2, MGAT2, and MGAT3B to play a role in NK cell escape in the absence of SPPL3 [15]. These enzymes are proteolytic targets of SPPL3 and catalyze *N*‐acetyllactosamine extension of complex *N*‐glycans, which are similar to the glycans present on nsGSLs. CD146 is such a protein harboring poly‐*N*‐acetyllactosamine chains, which, to our surprise, was better recognized by antibodies in the absence of SPPL3 [29, 30, 31]. Loss of SPPL3 highly disturbs the glycosylation landscape directly through upregulated glycosyltransferase expression and indirectly through limited availability of individual glycan precursor [15, 32, 33]. It is therefore conceivable that CD146 is hypo‐ or hyper‐glycosylated in the absence of SPPL3, which may improve specific antibody binding depending on the recognized CD146 epitope(s). Whether CD146 on target cells plays a role in NK cell‐mediated kill is unknown.

Together, regulation of complex *N*‐glycosylation, rather than glycosylation of GSLs, by SPPL3 contributes to sensitivity of tumor cells to NK cell activity.

Paradoxically, binding of NK cell‐activating receptor NKG2D to HAP1 cells improved with the loss of SPPL3 through a mechanism independent of nsGSLs. It is possible that other important ligand–receptor interactions are impeded which may favor tumor survival by sustaining the inactivation of NK cells in the functional assays. The diminished NKG2D‐Fc binding on HAP1 cells may have involved shedding of its ligands. Tumors use shedding of the ectodomains of NKG2D ligands MICA/B and ULPBs as an effective mechanism to escape from NK cell recognition [34, 35]. Shedding of these proteins involves activity of metalloproteases such as a disintegrin and metalloproteinase 10 (ADAM10) [36]. SPPL3 has also been shown to act as a selective sheddase that catalyzes the release of the ectodomain of substrates [32, 37, 38]. In addition, SPPL3 can mediate activation of metalloproteinase ADAM10 [39]. It is unknown if MIC proteins or ULBPs are direct substrates for SPPL3, though it could explain the increased binding of NKG2D when loss of SPPL3 would impair shedding and result in increased protein availability on the cell membrane.

In addition to the regulation of innate immunity through *N*‐glycosylation, regulation of nsGSL levels by SPPL3 impacted the trogocytosis by neutrophil‐like NB4 cells, underscoring the versatile mechanisms by which SPPL3 supports innate immune escape. In this work, we assessed neutrophil functionality using an established trogocytosis assay that utilizes the NB4 cell line. Repeating these assays with primary neutrophils or assessing neutrophil activity using conventional assays could provide additional insights into the effects of SPPL3 on innate immune responses.

Rigau and team identified SPPL3 in a genome‐wide knockdown screen as one of the key factors involved in Vδ2 TCR tetramer binding [40]. Here, we identify the involvement of SPPL3 in the actual killing of tumor cells by γδ T cells, which was mediated by downstream nsGSL synthesis as additional knockout of B3GNT5 reestablishes killing. The relation between SPPL3 and γδ TCR engagement is yet to be elucidated, although it is not unthinkable that nsGSLs shield butyrophilins comparable to HLA‐I and that this interaction contributes to the sum of all interactions.

Our previous work illustrated different degrees of impaired binding due to elevated nsGSLs for different antibodies targeting HLA [11]. We suggested that this was related to the position of the antibodies, as antibodies targeting the α3 domain (membrane proximal) were more impaired than antibodies binding epitopes on the α1 and α2 (peptide‐binding groove, membrane distal). In this study, we confirmed that position of the epitope plays a role by using a large set of antibodies. Comparison of proteins harboring epitopes that were affected by elevated nsGSL levels or not showed that proteins with relatively small ECD were prone to be affected over proteins with a larger ECD. With the exception of HLA‐I, the exact amino acids involved in binding of the antibodies used here are unknown. Future epitope mapping and affinity determination of these antibodies may allow generation of more complete analyses of the nsGSL‐mediated shielding phenomenon.

In addition, we utilized 13 different antibodies targeting CD147 for which we also had affinity information. The data generated using these antibodies showed that within affected regions of the protein, affinity could be a main determinant for the degree of shielding. Still, precise affinity measurements of the previously used HLA‐I‐specific antibodies or precise epitope identification of the CD147‐specific antibodies could provide an even better understanding on the role of affinity and the distance of these epitopes to the cell membrane in protein–protein (receptor–ligand) interactions in the presence of elevated nsGSL levels. Currently, however, the CD147 data represent only a single example. Our hypothesis on the basis of the CD147 data should be further validated with other natural or artificial proteins.

For the latter, one could design Ig‐domain‐containing scaffold proteins on which at different distances from the membrane, the same antibody epitope is engineered [41]. Different affinity versions of antibodies against such epitope could more definitively untangle antibody affinity and distance from the cell membrane [42], although additional mechanisms may further contribute to nsGSL shielding. One such parameter could be the outer charge of the cell surface receptor given the negative sialic acids are a requirement for nsGSL‐mediated shielding [11].

In our study, we found that protein expression by HAP1 WT and SPPL3^−/−^ is similar. Only the protein GSTM3 was not detected in SPPL3^−/−^ cells. This protein is an enzyme involved in the detoxification of compounds through the addition of a glutathione and is active in a different cellular compartment than SPPL3 [43]. GSTM3 is not described to be involved in the recognition of tumor cells by immune cells, nor is there a known link to SPPL3.

The work demonstrated here and by others indicates that SPPL3 is an essential regulator of membrane receptors functions through regulation of nsGSL synthesis and *N*‐glycosylation. Although only a fraction of membrane proteins was examined in this study, the selection process was random, suggesting that it could be representative for the majority of membrane proteins. Cell–cell interactions encompass a multitude of protein–protein interactions and the induction of multiple signaling pathways, culminating in cellular responses that hinge on the collective impact of these signals. It is not possible to assess the contribution of individual protein interactions to the described cellular immune responses, yet the cumulative interactome changes after loss of SPPL3 expression cause reduced reactivity by NK cells, γδ T cells, and neutrophils. However, targeting SPPL3 in therapies presents challenges due to its broad inhibitory function on glycosylation of multiple entities. Nevertheless, part of the regulation of SPPL3 goes through GSLs which could be targeted via more specific inhibitors. Enhancing the effectiveness of both cell‐ and antibody‐based therapies against nsGSL‐expressing tumors could be achieved by specific inhibitors of GSL synthesis such as Eliglustat or Miglustat (UGCG inhibitors) which are currently safely applied to patients with lysosomal storage disorders [11].

## Author Contributions

Tamara Verkerk performed research, analyzed the data, designed the study, and wrote the manuscript. Antonius A. de Waard performed research, analyzed the data, and contributed to the study design. Sofie J. I. Koomen, Jasper Sanders, Tineke Jorritsma, Anouk T. Pappot, Arie J. Hoogendijk, Floris P. J. van Alphen, and Tao Zhang performed research and analyzed the data. Nordin D. Zandhuis performed research and contributed to the design of the study. Manfred Wuhrer and Maartje van den Biggelaar provided advise on the study and data analysis. Hannes S. J. Stockinger provided reagents. Klaas P. J. M. van Gisbergen contributed to the study design and supervision of the study. Robbert M. Spaapen designed and supervised the study and wrote the manuscript. S. Marieke van Ham supervised the study and wrote the manuscript. All authors approved the final manuscript.

## Conflicts of Interest

The authors declare no conflicts of interest.

### Peer Review

The peer review history for this article is available at https://publons.com/publon/10.1002/eji.202451129.

## Supporting information



Supporting Information

## Data Availability

The data that support the findings of this study are available from the corresponding author upon reasonable request.
