# Supplementary material for: Tumor‐Expressed SPPL3 Supports Innate Antitumor Immune Responses
Source: Eur J Immunol. 2024 Dec 10;55(2):e202451129. doi: 10.1002/eji.202451129 (PMC11830388; doi:10.1002/eji.202451129)
Supplement: Supplementary file 1 — Supporting Information [file EJI-55-e202451129-s001.pdf]

## Supplemental tables

**Supplemental table 1:** Overview of antibodies and membrane dyes used for flow cytometry.

| Antibody | Clone | Conjugate | Dilution or concentration | Supplier | Reference |
|---|---|---|---|---|---|
| Anti-human TCR Vδ2 | B6 | PE | 1:200 | BioLegend | 331408 |
| Anti-human CD16 | 3G8 | BV605 | 1:500 | BioLegend | 302040 |
| Anti-human CD56 | NCAM16.2 | BV510 | 1:100 | BD Horizon | 563041 |
| Anti-human CD3 | UCHT1 | BV510 | 1:200 | BD Horizon | 563109 |
| Anti-human CD25 | 2A3 | BV605 | 1:100 | BD Horizon | 562660 |
| Anti-human CD69 | FN50 | BUV395 | 1:100 | BD Biosciences | 564364 |
| Anti-human/mouse Granzyme B | GB11 | FITC | 1:100 | BD Biosciences | 560211 |
| Anti-HLA-I/MHC-I | W6/32 | PerCP-eFluor 710 | 1:250 | Invitrogen | 46-9983-42 |
| Anti-human CD11b | Bear1 | PE | 1:100 | Beckman Coulter | IM2581U |
| Anti-human ICAM1 | 15.2 | AF546 | 1:10 (saturating)<br>1:100 (non-saturating) | Santa Cruz | Sc-107<br>AF546 |
| Goat anti mouse IgG | polyclonal | AF647 | 1:200 | Invitrogen | A21236 |
| Goat anti mouse IgG | M1310G05 | APC | 1:200 | Biolegend | 410712 |
| LIVE/DEAD$^{TM}$ Fixable Near-IR | | | 1:800 | Invitrogen | L34976A |
| VPD450 | | | 1:400 (1,0 mg/ml stock) | BD Horizon | 562158 |
| DiO lipophilic membrane dye | | | 1:200 (5 μM stock) | Invitrogen | V22886 |
| DIL stain | | | 1:400 (2,5 mg/ml stock) | Invitrogen | D3911 |
| AF350 NHS Ester | | | 1:100 (1,0 mg/ml stock) | Invitrogen | A10168 |
| Cell trace violet | | | 1:400 (1 μM stock) | Thermo Fisher Scientific | C34557 |
| CFSE | | | 125nM | Invitrogen | C34554 |

**Supplemental table 2:** Antibody panel figure 5 and 6.

| Target | Clone | Conjugate | Source | Reference |
|---|---|---|---|---|
| CD9 | FMC56 | | Leucocyte typing workshop | [21–23] |
| | CLB-thromb/8 | | Leucocyte typing workshop | [21–23] |
| | Gi7 | | Leucocyte typing workshop | [21–23] |
| | TP82 | | Leucocyte typing workshop | [21–23] |
| | PHN200 | | Leucocyte typing workshop | [21–23] |
| | MEM-61 | | Leucocyte typing workshop | [21–23] |
| | MB16 | | Leucocyte typing workshop | [21–23] |
| | BU-16 | | Leucocyte typing workshop | [21–23] |
| | BA2 | | Leucocyte typing workshop | [21–23] |
| | MM2/356 | | Leucocyte typing workshop | [21–23] |
| | M-L13 | | Leucocyte typing workshop | [21–23] |
| | TI117 | | Leucocyte typing workshop | [21–23] |
| | DS21 | | Leucocyte typing workshop | [21–23] |
| | 1AA2.H9 | | Leucocyte typing workshop | [21–23] |
| | GR2110 | | Leucocyte typing workshop | [21–23] |
| | MEM-192 | | Leucocyte typing workshop | [21–23] |
| | SN4 | | Leucocyte typing workshop | [21–23] |
| | PHN33 | | Leucocyte typing workshop | [21–23] |
| | ALB6 | FITC | Beckman Coulter | IM1755U |
| | DU-ALL1 | | Leucocyte typing workshop | [21–23] |
| | SN4d | | Leucocyte typing workshop | [21–23] |
| | Li 9.4 | | Leucocyte typing workshop | [21–23] |
| | MB61 | | Leucocyte typing workshop | [21–23] |
| CD13 | L138 | PE | BD | 347406 |
| | JP34-10 | | Leucocyte typing workshop | [21–23] |
| | Tük1 | | Leucocyte typing workshop | [21–23] |
| | MoU48 | | Leucocyte typing workshop | [21–23] |
| | CLB-mono-gran-2 | | Leucocyte typing workshop | [21–23] |
| CD15 | GF.26.7.3 | | Leucocyte typing workshop | [21–23] |
| | VIM10 | | Leucocyte typing workshop | [21–23] |
| | U1-4B4 | | Leucocyte typing workshop | [21–23] |
| | U1-58 | | Leucocyte typing workshop | [21–23] |
| CD29 | K20 | FITC | Beckman Coulter | IM0791U |
| | A-1A5 | | Leucocyte typing workshop | [21–23] |
| | JBS5 | | Leucocyte typing workshop | [21–23] |

| | | | | |
|---|---|---|---|---|
| | 4B4 | | Leucocyte typing workshop | [21–23] |
| | 13 | | Leucocyte typing workshop | [21–23] |
| CD36 | 131 | | Leucocyte typing workshop | [21–23] |
| | CLB-IVC7 | FITC | CLB | M1613 |
| | L103 | | Leucocyte typing workshop | [21–23] |
| CD46 | 46-1 | | In house produced (complement group) | |
| | 46-2 | | In house produced (complement group) | |
| | 46-3 | | In house produced (complement group) | |
| | 46-4 | | In house produced (complement group) | |
| | 46-5 | | In house produced (complement group) | |
| | 46-6 | | In house produced (complement group) | |
| | 46-7 | | In house produced (complement group) | |
| | 46-8 | | In house produced (complement group) | |
| | 46-9 | | In house produced (complement group) | |
| | 46-10 | | In house produced (complement group) | |
| | 46-11 | | In house produced (complement group) | |
| | GB24 | | In house produced (complement group) | |
| CD47 | B6H12 | | Leucocyte typing workshop | [21–23] |
| | BRIC125 | | Leucocyte typing workshop | [21–23] |
| | MP30-1, UMRh | | Leucocyte typing workshop | [21–23] |
| | CC2C6 | FITC | Biolegend | 323106 |
| | BRIC126 | | Leucocyte typing workshop | [21–23] |
| | 2D3 | FITC | Thermo Fisher Scientific | 11-0478-42 |
| CD49b | 6F1 | | Leucocyte typing workshop | [21–23] |
| | Gi9 | | Leucocyte typing workshop | [21–23] |
| | CLB-thromb/4, 10G11 | | Pelicluster | M1540 |
| | AK-7 | | Biolegend | 314306 |
| | HAS3 | | Leucocyte typing workshop | [21–23] |
| | 5e8 | | Leucocyte typing workshop | [21–23] |
| | Gi14 | | Leucocyte typing workshop | [21–23] |
| CD49d | HP1/7 | | Leucocyte typing workshop | [21–23] |
| | 8F2 | | Leucocyte typing workshop | [21–23] |
| | 9F10 | APC | BD | 559881 |
| CD49e | X6 | | Leucocyte typing workshop | [21–23] |
| | 3D3 | | Leucocyte typing workshop | [21–23] |
| | 2H6 | | Leucocyte typing workshop | [21–23] |
| | SAM1 | FITC | Beckman Coulter | IM1854U |
| | P4-7 | | Leucocyte typing workshop | [21–23] |
| | 16 | | Leucocyte typing workshop | [21–23] |
| CD49f | BQ16 | | Leucocyte typing workshop | [21–23] |
| | D1:EF | | Leucocyte typing workshop | [21–23] |
| | J8H | | Leucocyte typing workshop | [21–23] |
| | 135-13C | | Leucocyte typing workshop | [21–23] |
| CD51 | AMF7 | | Leucocyte typing workshop | [21–23] |
| | NKI-M9 | | Leucocyte typing workshop | [21–23] |
| | LM142 | | Leucocyte typing workshop | [21–23] |
| | NKI-M7 | | Leucocyte typing workshop | [21–23] |
| | 13C2 | | Leucocyte typing workshop | [21–23] |
| CD55 | BRIC110 | FITC | CLB | M2192 |
| | CD55 1 | | In house produced (complement group) | |
| | CD55 2 | | In house produced (complement group) | |
| | CD55-3 | | In house produced (complement group) | |
| | CD55-4 | | In house produced (complement group) | |
| | CD55-5 | | In house produced (complement group) | |
| CD59 | VJ1/12.2 | Pacific blue | Abcam | AB117734 |
| | 6H3 | | Leucocyte typing workshop | [21–23] |
| | MEM43 | FITC | CLB | M2191 |
| | OV9A2 | PE | eBioscience | 12-0596-42 |
| | MEM43 | | Leucocyte typing workshop | [21–23] |
| | p282 (H19) | BV421 | BD Biosciences | 564329 |
| CD70 | HNE.51 | FITC | DAKO | F7204 |
| | HNC.142 | | Leucocyte typing workshop | [21–23] |
| | CLB-2F2 | FITC | CLB | M1705 |
| | Ki-24 | PE | BD | 555835 |

| | | | | |
|---|---|---|---|---|
| | LD6 | | Leucocyte typing workshop | [21–23] |
| CD81 | 1D6 | | Leucocyte typing workshop | [21–23] |
| | 5A6 | | Leucocyte typing workshop | [21–23] |
| CD92 | VIM15b | | Leucocyte typing workshop | [21–23] |
| | VIM15 | | Leucocyte typing workshop | [21–23] |
| CD98 | GRVI | | Leucocyte typing workshop | [21–23] |
| | 4F2 | | Leucocyte typing workshop | [21–23] |
| CD146 | P1H12 | PE | BD | 550315 |
| | F432G-3 | | Leucocyte typing workshop | [21–23] |
| | F439E10 | | Leucocyte typing workshop | [21–23] |
| CD147 | UM-8D6 | | Leucocyte typing workshop | [21–23] |
| CD151 | 14A2 H1 | | Leucocyte typing workshop | [21–23] |
| | 11B1 G4 | | Leucocyte typing workshop | [21–23] |
| CD155 | D171 | | Leucocyte typing workshop | [21–23] |
| CD172a (SIRPa) | 12C4 | | In house produced | [44] |
| | 4G5 | | | |
| | 602411 | | R&D systems | MAB4546 |
| | 72 | | In house produced | |
| CD236 | NaM19-3C4 | | Leucocyte typing workshop | [21–23] |
| | BRAC1 | | Leucocyte typing workshop | [21–23] |
| | RB8 | | Leucocyte typing workshop | [21–23] |
| | BRIC10 | | Leucocyte typing workshop | [21–23] |
| | RB8 | | Leucocyte typing workshop | [21–23] |
| | BRIC4 | | Leucocyte typing workshop | [21–23] |
| CD321 | F11 | | Leucocyte typing workshop | [21–23] |
| HLA-I (MHC-I) | BB7.2 | APC | eBioscience | 17-9876-41 |
| | B1.23.2 | APC | eBioscience | 17-5935-42 |
| | W6/32 | PerCP-efluro710 | eBioscience | 46-9983-42 |

## Supplemental tables

Supplemental Figures

Supplemental Figure 1

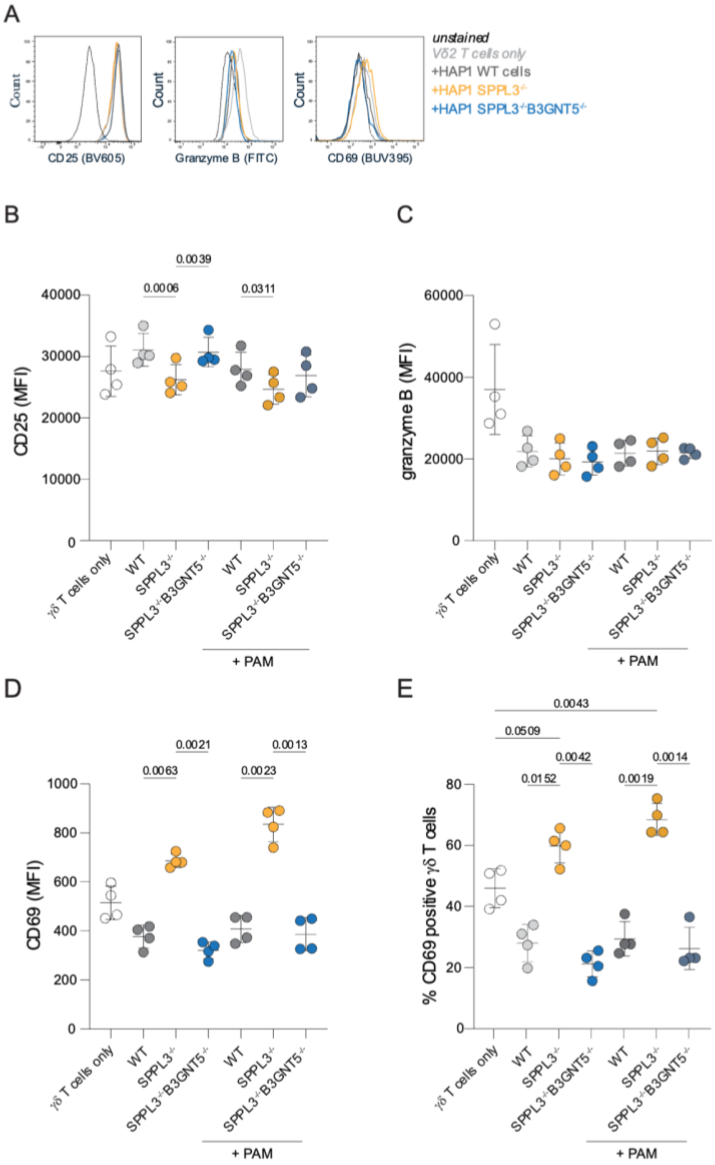


**Supplemental figure 1. Coculture of NK and γδ T cells with WT or SPPL3$^{-/-}$ tumor cells.**
(**A**) Histograms showing expression of CD25, granzyme B and CD69 by the γδ T cells after coculture with the HAP1 target cells. The MFI of CD25, granzyme B and CD69 positive γδ T cells **(B and C)** and percentage of CD69 positive cells **(E)** after coculture with HAP1 WT, SPPL3$^{-/-}$ or SPPL3$^{-/-}$ B3GNT5$^{-/-}$ cells. Data shown are representative from four experiments with four donors per experiment and three technical replicates per datapoint. A one-way ANOVA was used to assess statistical significances.

Supplemental Figure 2

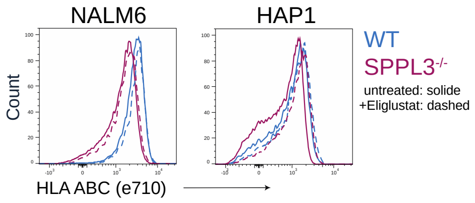


**Supplemental figure 2. HLA-I staining of NALM6 cells treated with a UGCG inhibitor**
Histograms of HAP1 and NALM6 WT (blue) and SPPL3$^{-/-}$ (yellow) cells stained for HLA-I either untreated (dashed line) or treated with UGCG inhibitor Eliglustat (solid line) for three days. Data shown represent two different batches and three technical replicates.

Supplemental Figure 3

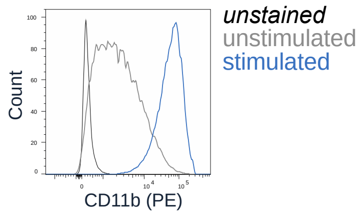


**Supplemental figure 3. CD11b expression by maturated NB4 cells.**
Flow cytometry plot demonstrating the CD11b staining of NB4 cells after seven days of stimulation with ATRA. Data shown represent four experiments with two technical replicates.

Supplemental Figure 4

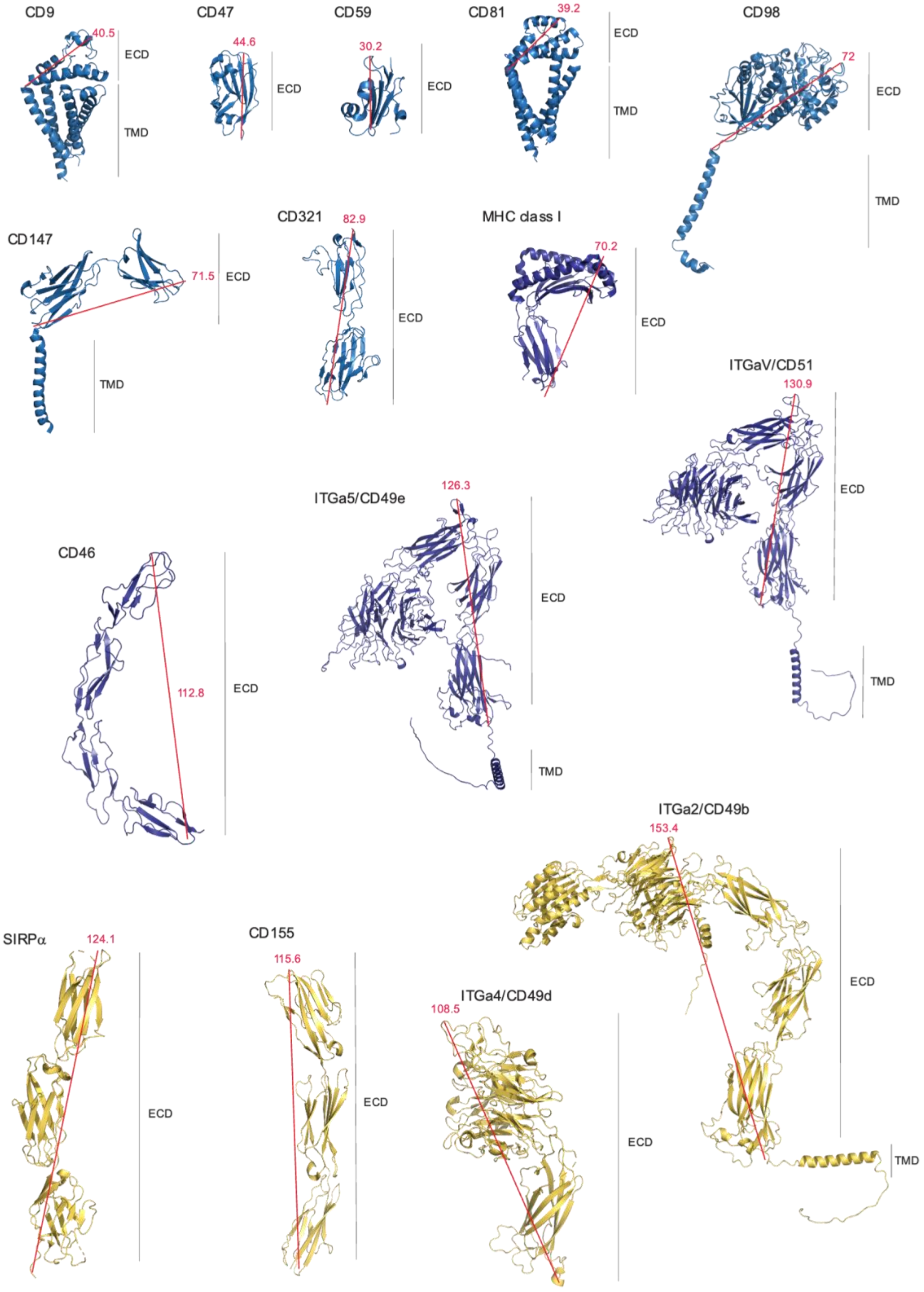

**Supplemental figure 4. Protein structures of proteins with and without epitopes identified to be affected by nsGSLs.**

Available protein structures were obtained from the Alphafold database for the proteins that contained epitopes that were affected (blue), for which at least one antibody was affected (purple) or not affected (yellow) by nsGSLs. The distance between the alpha carbon of the first amino acid of the extracellular domain to all other amino acids were determined in Armstrong (Å) using PyMOL and the longest theoretical distance is shown (red line). ECD: extracellular domain. TMD: transmembrane domain.

Supplemental Figure 5

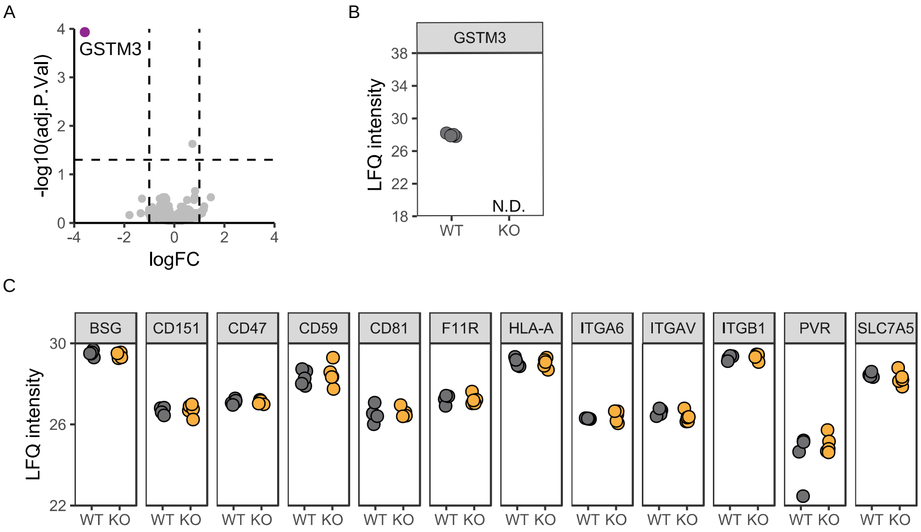


**Supplemental figure 5. Analysis of the protein abundance of HAP1 WT and SPPL3$^{-/-}$ cells from LC-MS/MS.**

(**A**) Differentially abundant proteins (FDR < 0.05 & |log$_2$ fold change| > 1, *n* = 5) between HAP1 WT and SPPL3$^{-/-}$ cells. (**B**) LFQ intensity for GSTM3. (**C**) LFQ intensities for detected proteins from the antibody panel shown in figure 5. Differential abundance (log$_2$ fold change) is plotted against the adjusted p-value (–log10 values). Data shown represent one experiment with five biological replicates.

Supplemental Figure 6

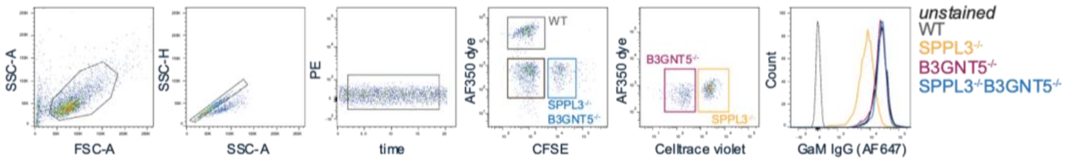


**Supplemental figure 6. Gating strategy**
HAP1 WT, SPPL3$^{-/-}$ and SPPL3$^{-/-}$B3GNT5$^{-/-}$ cells were given a membrane dye, mixed and incubated with CD147 targeting antibodies.
